# Patterns of neural activity in response to threatening faces are predictive of autistic traits: modulatory effects of oxytocin receptor genotype

**DOI:** 10.1038/s41398-024-02889-w

**Published:** 2024-03-29

**Authors:** Xiaoxiao Zheng, Feng Zhou, Meina Fu, Lei Xu, Jiayuan Wang, Jialin Li, Keshuang Li, Cornelia Sindermann, Christian Montag, Benjamin Becker, Yang Zhan, Keith M. Kendrick

**Affiliations:** 1 The Clinical Hospital of Chengdu Brain Science Institute, MOE Key Laboratory for NeuroInformation, University of Electronic Science and Technology of China, Chengdu, Sichuan China; 2 Brain Cognition and Brain Disease Institute (BCBDI), Shenzhen Institutes of Advanced Technology, Chinese Academy of Sciences, Shenzhen, China; 3 Southwest University, Chongqing, China; 4 Sichuan Normal University, Chengdu, Sichuan China; 5 University of Stuttgart, Computational Digital Psychology, Interchange Forum for Reflecting on Intelligent Systems, Stuttgart, Germany; 6 Department of Molecular Psychology, Institute of Psychology and Education, Ulm University, Ulm, Germany; 7 State Key Laboratory of Brain and Cognitive Sciences, The University of Hongkong, Hongkong, China

**Keywords:** Molecular neuroscience, Biomarkers

## Abstract

Autistic individuals generally demonstrate impaired emotion recognition but it is unclear whether effects are emotion-specific or influenced by oxytocin receptor (OXTR) genotype. Here we implemented a dimensional approach using an implicit emotion recognition task together with functional MRI in a large cohort of neurotypical adult participants (*N* = 255, male = 131, aged 17–29 years) to establish associations between autistic traits and neural and behavioral responses to specific face emotions, together with modulatory effects of OXTR genotype. A searchlight-based multivariate pattern analysis (MVPA) revealed an extensive network of frontal, basal ganglia, cingulate and limbic regions exhibiting significant predictability for autistic traits from patterns of responses to angry relative to neutral expression faces. Functional connectivity analyses revealed a genotype interaction (OXTR SNPs rs2254298, rs2268491) for coupling between the orbitofrontal cortex and mid-cingulate during angry expression processing, with a negative association between coupling and autistic traits in the risk-allele group and a positive one in the non-risk allele group. Overall, results indicate extensive emotion-specific associations primarily between patterns of neural responses to angry faces and autistic traits in regions processing motivation, reward and salience but not in early visual processing. Functional connections between these identified regions were not only associated with autistic traits but also influenced by OXTR genotype. Thus, altered patterns of neural responses to threatening faces may be a potential biomarker for autistic symptoms although modulatory influences of OXTR genotype need to be taken into account.

## Introduction

Individuals with autism spectrum disorder (ASD) typically exhibit general problems in discriminating and responding appropriately to face emotions [1–3], although it is unclear whether problems are generalized or emotion-specific [4, 5]. While several meta-analyses demonstrate impaired recognition across all face emotions in ASD [1, 3], findings are not consistent, with some studies reporting problems with only negative [6, 7] or positive [8] emotions while others have found no evidence for impairments [9–12]. This may reflect differences in tasks and the analytical approaches used [4, 5]. Additionally, a recent large study has reported that face emotion recognition impairments in ASD may be primarily due to a low functioning sub-group [13].

Face emotion recognition engages diverse psychological processes involving both subcortical and cortical circuitry [14–16]. Studies on face emotion processing distinguish a ‘core system’ implicated in visual processing and an ‘extended system’ for cognitive functions. The ‘core system’ is comprised of occipito-temporal regions while the ‘extended system’ includes the parietal cortex, orbitofrontal cortex (OFC), inferior frontal gyrus (IFG), medial prefrontal cortex (mPFC), amygdala, insula, anterior cingulate cortex (ACC), ventral striatum, and basal ganglia [14, 15]. Both ‘core’ and ‘extended’ processing systems exhibit atypical responses during face emotion recognition in ASD, with hypo- and hyper-activation being reported to all or specific emotional faces, particularly in the amygdala, fusiform face area, superior temporal sulcus and mPFC [13, 17–21]. On the other hand, some studies have found no differences in neural responses to emotional faces in ASD [10, 22] or in only in a sub-type of ASD individuals who also showed low face emotion recognition abilities and more severe symptoms [13].

The variability in both behavioral and neuroimaging findings for emotional face processing impairment in ASD may reflect the large heterogeneity in this disorder as well as task and analytical differences. For example, while the estimated heritability of ASD is around 60–70%, over 200 specific risk genes identified [23, 24] and heterogeneity in neural responses to social or other stimuli in ASD may therefore be contributed to by differences in genotypic as well as experiential contributions. In recent years there has been increasing interest in the role of the hypothalamic neuropeptide oxytocin in social cognition and ASD [25] with a number of studies having reported modulation of face emotion processing following intranasal administration of oxytocin in neurotypical individuals as well as those with ASD [25–27]. Peripheral oxytocin concentrations are decreased in children with ASD [28], and some clinical trials have reported improved social symptoms following chronic treatment with intranasal oxytocin [27, 29–31] and increased visual attention towards emotional faces [27]. A number of single nucleotide polymorphisms (SNPs) of the oxytocin receptor (OXTR) are associated with ASD symptoms, and risk genes identified as contributing to ASD influence oxytocin signaling [32, 33]. Evidence has repeatedly linked the polymorphisms of the OXTR gene, particularly the rs2254298, rs2268491, rs2268498, and rs53576 SNPs with autism, empathy, and social and emotional processing [34–42]. The OXTR rs53576 SNP is also associated with general sociability [43] and variations in rs2254298 may represent a trans-diagnostic biomarker for social dysfunction [44]. Finally, reduced amygdala and arousal responses to intranasal oxytocin are modulated by OXTR genotype (both rs53576 and rs22542980) [45].

Given the variability of both behavioral and neural findings reported by studies in clinical populations of ASD individuals in relation to emotion processing, and the likely contribution that the well-known heterogeneity of such individuals diagnosed with this disorder could have, the current study aimed to utilize a Research Domain Criteria (RDoc) inspired dimensional approach [46]. To this end we aimed to establish firstly whether autistic traits per se in a large sample of neurotypical individuals (*n* = 255), not diagnosed with ASD, are associated with general or emotion-specific behavioral and neural responses to face expressions (angry, fear, happy, neutral and sad) and secondly if there are modulatory influences of their OXTR genotype. For the face emotion recognition task we used an implicit task approach where participants only had to identify the sex of individual displayed to help avoid participants trying to consciously identify face emotions during presentations in the MRI scanner rather than simply process face emotion stimuli. By including a simple requirement to identify the sex of the face presented we ensured that participants paid attention to the stimuli. Explicit face emotion recognition, where participants were required to actually identify specific face emotions, was also tested subsequently outside of the scanner to provide a measure of recognition accuracy and speed. Neuroimaging analyses based on univariate approaches have inherent limitations since they can only measure associations between activity changes for specific voxels or regions, so we therefore chose to use multivariate pattern analysis (MVPA) [47]. MVPA can decode the multivariate information contained in functional patterns using a classifier. By employing a searchlight strategy with machine learning it can determine differences in the patterns of activation exhibited which are predictive of specific features of individuals using the patterns of their neural responses to stimuli and without the potential bias of prior voxel selection [47]. We therefore used MVPA to extract information from locally distributed fMRI-based patterns of activation during performance of a face emotion task to permit a powerful fine-grained analysis of differences in extensive neural activation patterns which are predictive of autistic traits. This MVPA approach has been used in several previous studies comparing small numbers of ASD and neurotypical individuals [10, 22] but not in large scaled ones. Given that differences in patterns of neural responses across multiple brain regions identified by MVPA are likely to be influenced their functional connectivities, we additionally used a univariate analysis to investigate these. To assess potential modulatory effects of multiple OXTR genotypes on these ASQ-associated functional connectivities we then investigated whether the ASQ associations were modulated by individual OXTR genotype by examining the influence of four SNPs associated with autism and social cognition (rs2254298, rs2268491, rs2268498, rs53576) to determine whether autistic trait (Autism spectrum quotient (ASQ)) [48, 49] associations were genotype-dependent.

Overall, we hypothesized that there would be a negative association between autistic traits and accuracy in identifying the expressions but not the sex of emotional faces and that MVPA would reveal that there are patterns of activity in both ‘core’ and ‘extended’ face processing regions in response to specific emotions which are predictive of autistic trait scores. We also hypothesized further analysis would reveal that the strength of functional connectivity between regions identified by the MVPA as predictive of autistic trait scores would be associated with these scores in an OXTR genotype-dependent manner.

## Materials and methods

### Participants

255 neurotypical Han Chinese (male = 131; age range = 17–29 years, mean age $\pm$ SD = 21.62 $\pm$ 2.339 years) participants were enrolled (for inclusion criteria see Supplementary). Sample size was not pre-determined but post-hoc analysis showed that this sample size achieved >90% power for a medium effect size in all types of statistical tests used in the data analysis (calculated by G*Power v3.1.9.4). Participants reported being free from current or past medical, neurological, or psychiatric conditions, and no history of head injury or MRI contraindications. All volunteers were required to abstain from alcohol, caffeine-containing drinks, cigarettes or other psychoactive substances during the 24 h prior to the experiment. Before the experiment all participants provided written informed consent. The study was approved by the local ethics committee (Institutional Review Board, University of Electronic Science and Technology of China) and in accordance with the latest revision of the Declaration of Helsinki. The study was part of a large-scale fMRI project composed of multiple task-based paradigms investigating diverse questions including inhibitory control [50, 51], imitation and the mirror neuron system [52] and pain empathy [53, 54]. In contrast to these previous studies, the current one aimed to establish associations between autistic traits and neural and behavioral responses to specific face emotions, along with modulatory effects of OXTR genotype.

A total of 26 participants were excluded due to failure to complete the study (*n* = 6), head movement (*n* = 12; see fMRI data preprocessing for details) or technical failures (incomplete data, *n* = 8), leaving a final experimental cohort of 229 participants (males = 114).

### Experimental procedures

**Fig. 1:**
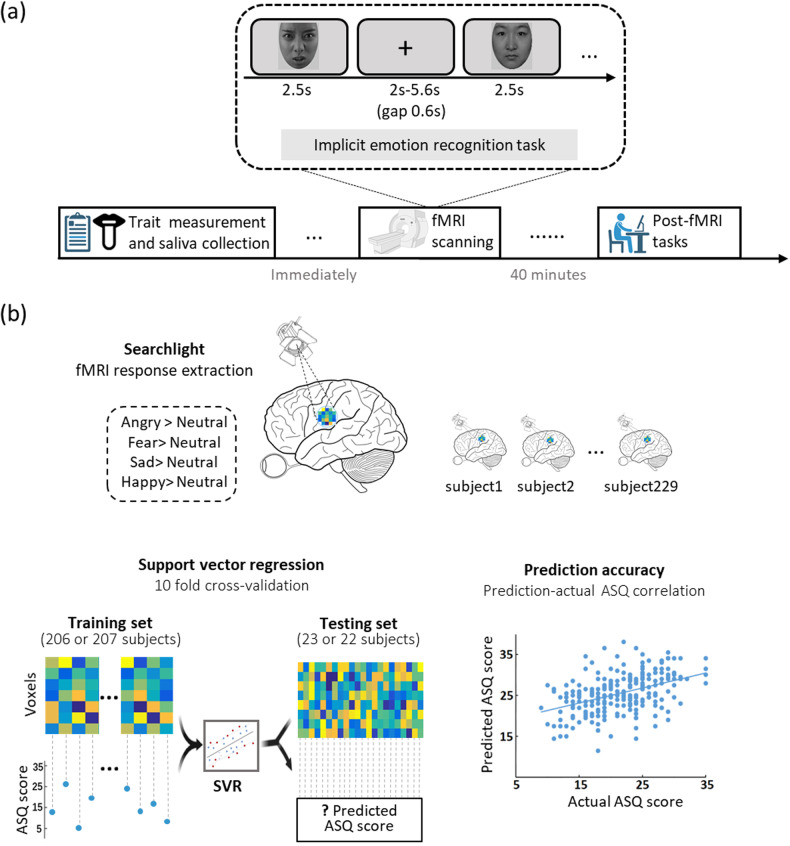
Experimental protocol and the searchlight-based multivariate pattern analysis (MVPA). **a** The experimental protocols and event-related implicit emotion recognition fMRI paradigm. **b** Illustration of the searchlight-based multivariate pattern analysis (MVPA). Three-voxel radius spherical searchlights around center voxels were employed for each contrast separately, with individual beta maps as features to predict participants’ ASQ scores. The optimal hyperplane was computed based on the multivariate pattern for 10 different iterations of a training cohort of a sub-set of participants and another excluded subset (test set). Regional activation patterns which could robustly predict autistic traits, pattern maps were corrected for multiple comparisons and a Pearson correlation analysis used to measure the correlation between actual and predicted ASQ scores and those predicted by the MVPA.

The experimental protocols are presented in Fig. 1a. Autistic traits were assessed by the ASQ [48, 49] with Cronbach’s α scores in the present sample being 0.744. The ASQ is a widely used measure of autistic traits in both neurotypical and ASD individuals and comprises 50 self-report questions. It is not intended as a diagnostic tool per se and total scores for the level of autistic traits are calculated from responses to questions in five different sub-domains (social skills, social communication, attention to detail, attention switching and imagination). Total ASQ scores range from 0 and 50 with higher scores representing greater autistic traits and a suggested clinical threshold of scores above 32 [48]. All participants provided buccal swaps for analysis of OXTR (rs2254298, rs2268491, rs2268498, rs53576) genotype (see Supplementary and [35]).

An event-related design implicit emotional face recognition task was implemented during fMRI scanning. Fifty grayscale facial stimuli displaying angry, fear, happy, neural or sad expressions (*n* = 10 per category, 50% male, each from different identities) were presented twice in two runs with a different pseudorandom sequence for all participants for discrimination of sex (see Fig. 1a for paradigm and Supplementary). About forty minutes after fMRI scanning participants completed a surprise explicit face recognition memory test to establish if they remembered the facial stimuli presented during fMRI. This was used to assess possible associations between autistic trait scores on the ASQ and accuracy and corresponding response times (RT), but was not used in the analysis of the previous fMRI responses (see supplementary and Fig. S1).

### MRI data acquisition and preprocessing

MRI data were acquired on a 3T GE MR750 system (General Electric, Milwaukee, WI, USA). High-resolution whole-brain T1-weighted structural MRI data were acquired to improve normalization of the functional images. Task-based fMRI data were acquired during the implicit face recognition task (lasted around 12 min, two sessions were implemented, each session consisted of 173 volumes). Functional MRI data were preprocessed using SPM12 (Statistical Parametric Mapping; http://www.fil.ion.ucl.ac.uk/spm) (see Supplementary for details).

### Analytic approach for behavioral data

During the fMRI implicit emotion recognition task, discrimination accuracy for the sex of faces and RT were calculated. For the post-fMRI test, recognition accuracy and RT for both the surprise face memory test and explicit emotion recognition task, as well as ratings of arousal and intensity were calculated. All RTs were calculated only for trials where accurate identification of face sex or emotion was shown. Repeated measures ANOVAs with emotion expression (angry, fear, sad, neutral, and happy) as the within-subject factor were conducted to explore the main effect of emotion expression on behavioral indices. To further investigate if autistic traits were associated with behavioral indices, correlational analyses (Pearson) were conducted.

### Analytic approach for MRI data

#### General linear model (GLM) analyses

First-level general linear models (GLMs) for the task-based fMRI data included condition-specific regressors of the 5 emotions (angry, fear, happy, neutral, and sad) were modelled to generate main contrasts of interest (angry > neutral, fear > neutral, happy > neutral, sad > neutral) and the six movement parameters were included as nuisance regressors.

#### Pattern-based MVPA analyses and thresholding

To determine if local activation patterns were predictive of autistic traits (ASQ scores), a whole-brain (restricted to a grey matter mask) searchlight-based multivariate machine-learning pattern analysis was implemented (see Fig. 1b for illustration). Specifically, we employed a support vector regression (SVR) algorithm implemented in the Spider toolbox (http://people.kyb.tuebingen.mpg.de/spider) (linear kernel, *C* = 1) with three-voxel radius spherical searchlights around center voxels employed for each contrast separately, with individual beta maps as features to predict participants’ ASQ scores. The accuracy with which the significant neural activation patterns in response to different face emotions identified by the MVPA could be predictive of actual ASQ scores was determined by a Pearson correlation analysis between the actual ASQ scores and those predicted by the MVPA. The prediction performance was evaluated by a tenfold cross-validation procedure during which all participants were randomly assigned to 10 subsamples of 22 or 23 participants using MATLAB’s cvpartition function. The optimal hyperplane was computed based on the multivariate pattern of the labeled 206 or 207 participants (training set) and then evaluated using the excluded 22 or 23 participants (test set). This procedure was repeated 10 times with each subsample being the testing set once. To identify regional activation patterns which could robustly predict autistic traits, pattern maps were corrected for multiple comparisons, within a grey matter mask based on false discovery rate (FDR) and additionally taking into account multiple comparisons involving the different face emotions (i.e. 4 contrasts) resulting in a corrected threshold at a whole-brain voxel level of *q* = 0.0125 FDR corrected (two-sided).

#### Functional connectivity (FC) analyses and thresholding

To further explore associations between autistic traits and FC in face-emotion processing networks, and modulatory effects of OXTR genotype, seed-to-whole-brain FC analyses with the contrasts exhibiting significant results from the MVPA analyses were computed using generalized psychophysiological interactions (gPPI) [55]. Seeds (6-mm spheres) were placed at peak coordinates of significant clusters from the MVPA analyses. FC analysis firstly employed a whole brain approach with a significance threshold of *p* < 0.05 peak-level family-wise error (FWE) correction and a minimum voxel size of *k* > 10. To take into account the three different face emotions for which the MVPA showed significant effects Bonferroni corrections (×3) were additionally applied. Brain regions were identified using the Automated Anatomic Labelling atlas 3 (AAL3) [56] as implemented in the WFU Pick Atlas (School of Medicine, Winston-Salem, North Carolina).

#### Influence of OXTR genotype

Seed-region-specific connectivity maps were entered into two-sample t-test models, with genotype group (i.e., rs2254298- A+ vs. A- carriers), ASQ score as well as their interaction terms as covariates. When significant genotype × ASQ interactions were found, parameter estimates from the significant clusters were extracted to visualize the interaction effects. Bonferroni corrections were applied for the number of SNPs and alleles (i.e. 4 × 2 = 8).

## Results

### Sample and genotyping

A total of 229 right-handed neurotypical Han Chinese participants (male = 114; mean age ± SD = 21.58 ± 2.343) were included in the final analysis (see Fig. S2 for exclusion flowchart). Distribution of the 4 OXTR (rs2254298, rs2268491, rs2268498, and rs53576) genotypes were in the Hardy-Weinberg equilibrium (HWE) and alleles of the SNPs were divided into two groups as in previous studies [39, 45] to increase statistical power and avoid statistical inference errors (see Table S1). Mean ± SD total scores on the ASQ were 21.44 ± 5.60 (range 9–35). These ASQ scores are in good agreement with our previous large-scale (*n* = 280) study on Chinese adult neurotypical individuals and there were also no significant differences in ASQ scores between males (21.56 ± 5.42, range 10–35) and females (21.32 ± 5.80, range 9-35) [37] (*t*-test *p* = 0.747).

### Behavioral results

**Fig. 2:**
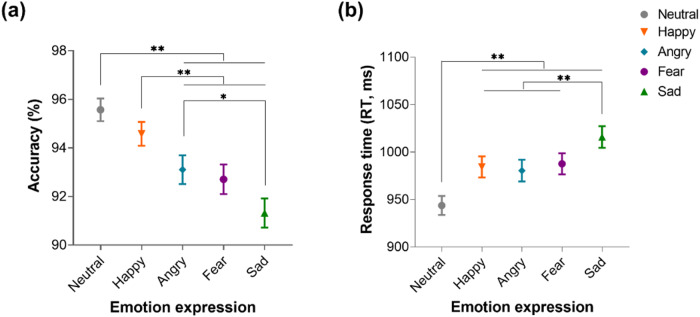
Behavioral results. Discrimination of face sex (**a**) accuracy rate and (**b**) response time (RT) during the implicit emotion recognition task during fMRI scanning. *p < 0.05, **p < 0.01.

Two participants were additionally excluded due to incomplete data for the implicit emotion recognition task, leading to 227 participants (male = 112; mean age $\pm$ SD = 21.57 $\pm$ 2.351 years) included for behavioral data analyses. ANOVA results for the implicit emotion recognition task showed a main effect of face emotion for both accuracy (F_(4,226)_ = 21.257, *p* < 0.01, *η*_*p*_^*2*^ = 0.086) and RTs (F_(4,214)_ = 24.747, *p* < 0.01, *η*_*p*_^*2*^ = 0.099). Post-hoc Bonferroni corrected tests revealed that participants were less accurate for discriminating the sex of individuals with negative emotional expressions (angry, fear, and sad) compared to neutral and happy faces (all *p*s < 0.049) (Fig. 2a). The RTs for all face emotions (angry, fear, sad and happy) were significantly longer than for neutral expressions (all *p*s < 0.002) (Fig. 2b). Overall these results suggest that participants attentively processed the facial stimuli (i.e. discriminated the sex of individual faces accurately) and that presentation of negative emotional faces resulted in a lower accuracy and longer RTs.

For the post fMRI emotion recognition memory task an ANOVA revealed a significant main effect of emotion expression (F_(4,226)_ = 24.359, *p* < 0.01, *η*_*p*_^*2*^ = 0.097) (F_(4,226)_ = 24.359, *p* < 0.01, *η*_*p*_^*2*^ = 0.097). Post-hoc Bonferroni-corrected comparisons showed participants remembered fearful, sad, and happy emotional faces better than neutral and angry ones, and fearful faces better than happy ones (see Supplementary Fig. S3). There was no main effect of emotion for RTs (F_(4,226)_ = 1.206, *p* = 0.305, *η*_*p*_^*2*^ = 0.005).

For emotion discrimination accuracy, an ANOVA revealed a significant main effect of emotion expression (F_(4,226)_ = 105.298, *p* < 0.01, *η*_*p*_^*2*^ = 0.318). Post-hoc Bonferroni-corrected comparison showed participants exhibited the highest discrimination accuracy for happy faces and the lowest for fearful faces. For RTs there was also a main effect of emotion expression (F_(4,226)_ = 140.011, *p* < 0.01, *η*_*p*_^*2*^ = 0.383) with the shortest RTs for neutral expression faces and the longest for fearful ones. For arousal and intensity ratings, ANOVAs revealed significant main effects of emotion expression (Arousal: F_(4,226)_ = 258.318, *p* < 0.01, *η*_*p*_^*2*^ = 0.533; Intensity: F_(4,226)_ = 319.985, *p* < 0.01, *η*_*p*_^*2*^ = 0.586) with post-hoc comparisons showing participants rated angry faces as the most arousing and intense with neutral ones as the least. Arousal ratings for sad faces were significantly lower compared with both fearful and happy ones. (see Supplementary Fig. S4).

However, no significant associations were found between explicit face emotion recognition accuracy or RTs or recognition memory for faces or arousal or intensity ratings and ASQ scores (see Supplementary).

### Findings from MVPA analyses

**Fig. 3:**
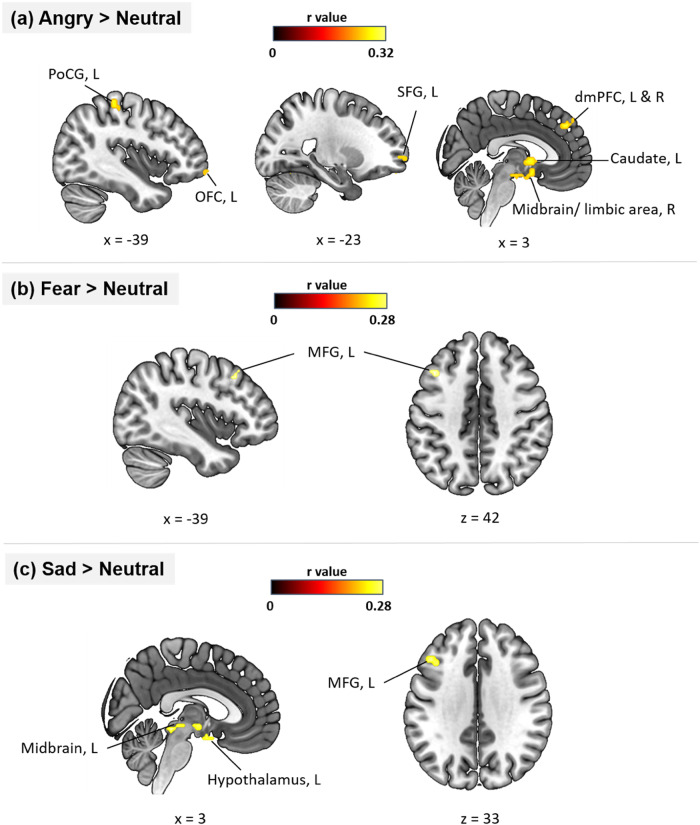
Results of MVPA analyses. Local brain regions that predict individual autistic traits revealed by MVPA analyses in **a** angry versus neutral, **b** fear versus neutral, and **c** sad versus neutral contrasts. Statistical significant results were thresholded at whole-brain voxel level FDR *p* < 0.0125 (two-sided). Color bar denotes prediction-outcome correlation. MVPA multivariate pattern analyses, dmPFC dorsal medial prefrontal cortex, MFG middle frontal gyrus, OFC orbitofrontal cortex, PoCG postcentral gyrus, SFG superior frontal gyrus, L left, R = right.

The SVR-based MVPA analysis revealed activity patterns of the right midbrain (extending into limbic areas) comprising the ventral tegmental area (VTA), anterior cingulate cortex (ACC), hypothalamus (MNIxyz = 3/−3/−18, *k* = 40), the left anterior orbitofrontal cortex (OFC) (MNIxyz = −39/57/−9, *k* = 11), left caudate (MNIxyz = −3/−6/0, *k* = 20), right dorsal medial frontal cortex (dmPFC; MNIxyz = 3/42/42, k = 12), left postcentral gyrus (PoCG; MNIxyz = −45/−33/51, *k* = 45), and the left superior frontal gyrus (SFG; MNIxyz = −21/63/0, *k* = 6) could accurately predict individual autistic traits during processing of angry (versus neutral) expressions (Fig. 3a). Additionally, during processing fear (versus neutral) expressions, individual autistic trait scores could be decoded from activity in the left MFG (MNI_xyz_ = −36/18/42, *k* = 8) (Fig. 3b). Individual autistic-trait scores could also be decoded from neural activity towards sad (versus neutral) expressions in the left MFG (MNI_xyz_ = −36/24/33, *k* = 7), the right midbrain (MNI_xyz_ = −3/−30/−9, *k* = 13), and left hypothalamus (MNI_xyz_ = −3/−3/−15, *k* = 30) (Fig. 3c).

### Analysis of FC associations with ASQ and influence of OXTR genotype

The gPPI analyses using a seed-to-whole brain approach (seeds defined according to the MVPA analyses) revealed no significant (*p*_FWE_ < 0.05) FCs associated with trait autism during processing of specific emotional faces irrespective of OXTR genotype. However, there were significant interaction effects of ASQ and genotype for rs2268491 and rs2254298 on FC during processing of angry expressions.

**Fig. 4:**
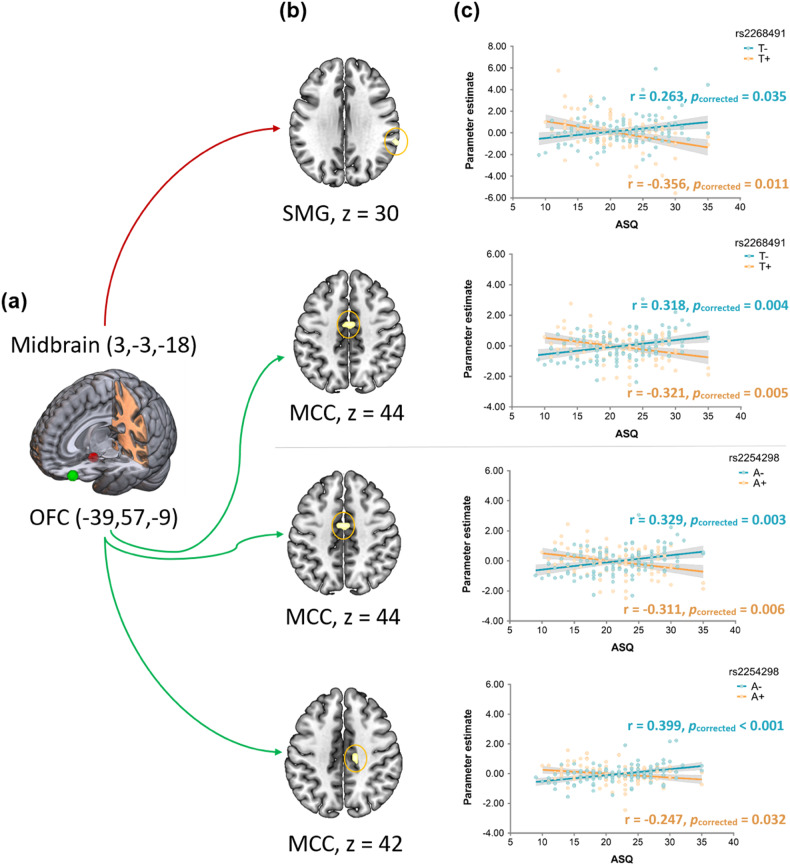
Interaction of autistic traits (ASQ) and OXTR genotype (rs2268491-top panel; rs2254298-bottom panel) on whole-brain FC during the process of angry versus neutral emotion. **a** Seeds of interest, i.e. Midbrain and OFC. **b** Regions exhibiting significant effect of autistic trait (measured by ASQ) on FC strength as a function of rs2268491 (Top panel) and rs2254298 (Bottom panel) genotypes respectively. **c** Parameter estimates from the significant interaction clusters extracted for visualization purpose. Statistical significance thresholding was applied with whole-brain peak level at *p*_FWE_ < 0.0125 and a minimum voxel size of *k* > 10 Bonferroni corrections (×3) were additionally applied for the three different face emotions with significant patterns identified by MVPA. The *p*-value for A+ rs2254298 is one-tailed. ASQ autism spectrum quotient, FC functional connectivity, OFC orbitofrontal cortex; MCC middle cingulate cortex, SMG supramarginal gyrus.

Examination of rs2268491 genotype (T+/T−) × ASQ score interactions revealed effects for the angry > neutral contrast in coupling of left OFC (seed, *x* = −39, *y* = 57, *z* = −9) with bilateral MCC (MNIxyz = 0/−9/45, *k* = 24, T = 5.11, *p*_FWE-peak_ = 0.005; *pcorrected for number of significant face expressions* = 0.015) (Table 1). Parameter estimates from the significant clusters extracted to visualize interaction effects found opposite effects of ASQ scores on FC strengths in the T+ and T- groups. Pearson tests (*p* values Bonferonni corrected for the number of SNPs and alleles i.e. 4 × 2 = 8) revealed a positive association between ASQ and the FC strength in the T- group and a negative association in the T+ group (T-: *r* = 0.318, *pcorrected* = 0.004; T+: *r* = −0.321, *pcorrected* = 0.005) (Fig. 4). Additionally, ASQ and rs2268491 interactively impacted FC strengths between the right midbrain (seed, x = 3, y = −3, z = −18) and right supramarginal gyrus (SMG) (MNIxyz = 63/−39/30, *k* = 14, *T* = 4.92, *p*_FWE-peak_ = 0.012, *pcorrected for number of significant face expressions* = *0.036)* (Table 1). Parameter estimates were subsequently extracted and showed a positive association between ASQ and the FC strength in the T− group but negative association in the T+ group (T−: *r* = 0.263, *pcorrected* for number of SNPs and alleles = 0.035; T+: *r* = −0.356, *pcorrected* = 0.011) (Fig. 4).

**Table 1:** FC modulated by OXTR genotype during processing of angry > neutral face emotion.

Region	*k*	*t*-value	*p*_FWE-0.05_	*p*_corrected_	*x*	*y*	*z*
*rs2254298*							
seed: OFC (−39/57/−9)							
MCC, R	14	5.10	0.006	0.018	15	−21	42
MCC, L/ MCC, R	23	5.05	0.007	0.021	0	−9	45
*rs2268491*							
seed: OFC (-39/57/-9)							
MCC, L/ MCC, R	24	5.11	0.005	0.015	0	−9	45
seed: Midbrain (3/−3/−18)							
SMG, R	14	4.92	0.012	0.036	63	−39	30

Statistical significance thresholding was applied with whole-brain peak level at *p*_FWE_ < 0.05 and a minimum voxel size of *k* > 10. Bonferroni corrections (x3) were additionally applied for the three different face emotions with significant patterns identified by MVPA.

*OFC* orbitofrontal cortex, *MCC* middle cingulate cortex, *SMG* supramarginal gyrus, *L* left, *R* right.

Significant interaction effects of genotype of rs2254298 (A+/A−) × ASQ scores were also observed during processing angry face emotion in FC between left OFC (seed, *x* = −39, *y* = 57, *z* = −9) and left MCC (extended to the right MCC) (MNIxyz = 0/−9/45, *k* = 23, *T* = 5.05, *p*_FWE-peak_ = 0.007, *pcorrected for number of significant face expressions* = 0.021), and additional clusters located in the right MCC (MNIxyz = 15/−21/42, *k* = 14, *T* = 5.10, *p*_FWE-peak_ = 0.006, *pcorrected* = 0.018) (Table 1). Parameter estimates were extracted and further revealed opposite effects of ASQ on these two couplings in A+ and A- carriers. ASQ was positively associated with the left OFC - left MCC coupling in A- carriers while A+ carriers exhibited a negative correlation (A-: *r* = 0.329, *pcorrected* for number of SNPs and alleles = 0.003; A+: *r* = −0.311, *pcorrected* = 0.006) (Fig. 4). Likewise, for A- carriers there was a positive correlation between autistic trait scores and left OFC - right MCC coupling but a negative correlation was in A+ carriers (A-: *r* = 0.399, *pcorrected* < 0.001; A+: *r* = −0.247, *pcorrected* = 0.032 one-tailed) (Fig. 4).

No significant interaction effects were observed for other seeds nor during processing fear or sad expressions. The analyses for SNPs rs53576 and rs2268498 did not yield any significant effects.

## Discussion

The current study aimed to identify the neural pattern predictive of autistic traits during processing of different emotional faces and potential modulatory effects of OXTR genotype. There were no associations between trait autism and either implicit or explicit face emotion recognition accuracy. However, the SVR-based MVPA identified activity patterns that strikingly predicted individual autistic traits during processing of angry emotional expressions in regions involved in emotion, reward and salience, but not in early visual, processing. As such, they indicate that higher autistic traits tend to impact the ‘extended’ rather than the ‘core’ face emotion processing system. A seed-based FC analyses revealed that two OXTR SNPs (rs2254298, rs2268491) showed a modulatory effect on OFC-MCC coupling during processing of angry faces. Overall, these findings indicate that trait autism is predicted from extensive altered patterns of neural activity and reduced responses during processing of angry expressions in motivation, reward and salience processing networks. The association between FC of the OFC and MCC and angry faces is also OXTR genotype-dependent.

Although we found overall evidence for negative emotional expressions influencing recognition accuracy of sex and emotion expression as well as arousal and intensity ratings, there was no evidence for associations between implicit or explicit face emotion recognition performance and ASQ scores. This is in line with a number of studies comparing autistic and neurotypical individuals [9–12], although other studies have either reported general impairment across face expressions or for negative or positive emotions in ASD [1, 3]. Overall, the absence of any significant association between face emotion recognition and trait autism may reflect our use of a dimensional approach, but it should also be noted that discrimination accuracies were very high (>90% except for fear), and associations with trait autism might have been revealed with an increased task difficulty (see [50]). Another possibility is that autistic traits have less of an effect on discrimination/decoding of emotional faces and more on subsequent cognitive and emotional processing. The absence of any associations between trait autism and responses to emotional faces in early visual processing regions may support this interpretation.

The SVR-based MVPA revealed whole-brain neural representative relevant for autistic traits across all negative face emotion expressions, although particularly for anger. Trait autism scores were predicted by patterns across a wide network of cortical and subcortical regions engaged in the ‘extended’ face emotion network including those involved in emotion, motivation, reward and salience processing. Predictive patterns were mainly found in limbic regions (hypothalamus), the social salience system (ACC) and most notably fronto-basal ganglia-midbrain reward systems (dmPFC, SFG, MFG, OFC, caudate and VTA). The MVPA thus revealed evidence for their spatial patterns of activity being predictive of dimensional autistic traits. Notably, the MVPA was not predictive of trait autism in the “core” occipital and temporal cortex face emotion processing systems. This supports the conclusion that later cognitive rather than early visual processing of angry faces is sensitive to autistic traits.

Diminished patterns of activation and responses to angry faces associated with higher autistic traits in limbic regions may indicate reduced processing of threatening emotional stimuli [57, 58]. However, our MVPA analysis only revealed hypothalamic rather than amygdala spatial patterns predicted autistic traits. The hypothalamus, is involved in emotion processing [59–62], can function as a valence detector and modulator [60, 63] and is highly connected with the hippocampus and amygdala [64–66]. Hypothalamic morpho-functional differences have also been reported in ASD [67].

The ACC in the brain salience network was also predictive of trait autism during the processing of angry faces suggesting that higher autistic trait individuals perceive threatening stimuli as less salient. The ACC plays a central role in processing subjective evaluation and emotional salience-associated with cognitive processing, executive control and self-awareness [68–72] and shows altered structure and function in ASD [72–74]. Two recent electroencephalography studies have also reported differences in cingulate and other cortical responses to angry faces in individuals with ASD both in terms of patterns of synchronization associated with symptom severity [75, 76] and evoked potentials in cingulate and other cortical regions, potentially indicating impaired salience processing.

The ability of patterns of activation in fronto-basal ganglia circuitry to predict autistic trait scores is in line with the “Dopamine Theory of ASD” [77, 78]. According to this theory, the dysfunctional midbrain dopaminergic system which projects to the prefrontal cortex and striatum via both mesocorticolimbic (MCL) and nigrostriatal (NS) circuits leads to impaired reward processing and motivation-related behavior along with altered goal-directed motor behavior and habitual behavior, contributing to core behavioral features of ASD. Although fronto-basal ganglia circuitry plays an important role in reward and motivation, it also transmits signals related to salient, but non-rewarding, experiences such as aversive and alerting events and thus additionally plays a crucial role in motivational control of either approach or avoidance behavior [79]. Individuals with ASD tend to have greater problems in recognizing angry expression faces rather than other emotions independent of alexythmia [80] and children with ASD exhibit an attentional bias away from angry faces at long presentation rates with greater avoidance being associated with greater social communication difficulties [81]. Thus, in ASD, the basic pre-dispositional mechanisms to allocate attention quickly towards angry faces may be weaker [82]. However, given that we only found patterns of activation in response to angry faces were predictive of autistic trait scores in the ‘extended’ rather than the ‘core’ face processing network this would tend to support the argument that greater autistic symptoms may primarily lead to altered interpretation and responses to angry faces rather than simply to reduced attention towards them. Indeed, in our behavioral analyses accuracy and response times for identifying the sex of face emotions did not reveal any problems specific to angry emotion faces, again suggesting that participants did pay attention to them. Thus, avoidance of angry faces in ASD may have more to do with post-attentive cognitive and motivational processing.

Our initial hypothesis that OXTR genotype would influence associations between functional connectivity in neural circuitry identified by the MVPA as predictive of autistic traits was supported, in line with previous findings [83]. The intrinsic FC of OFC-MCC and midbrain-SMG was dependent upon both autistic traits and OXTR genotype (either rs2254298 or rs2268491) for responses to angry faces. Carriers of the risk alleles of the SNPs (A+ or T+) associated with social cognition dysfunction and ASD [32, 39, 84] showed reduced FC in individuals with higher autistic trait scores but increased connectivity in those carrying the non-risk allele (A- or T-). The neural coupling of OFC to MCC may be involved in reward and motivation processing given that the MCC is known to play a critical role in both reward [58, 85–87] and emotion [58, 88] processing. Connectivity between midbrain and SMG may also play an important role in emotion processing [89], especially for the negative emotions [90, 91]. Both SMG and MCC are critical for social functioning in the context of ASD such as empathic processing [43, 92, 93] and self-other distinction [83, 94] and may contribute to representation and integration of internal and emotional feeling states. A previous study has also reported a genotype × autism symptom interaction for SMG responses during face emotion recognition for rs2254298, rs2268491and rs53576 OXTR SNPs [37]. The OXTR SNPs rs2268491and rs2254298 exhibit high linkage disequilibrium (LD) [32, 84] which may explain their common modulatory effects.

There are some limitations to be noted. Firstly, the study adopted a dimensional approach using only neurotypical individuals and measurement of trait autism using the ASQ and findings should be confirmed in a clinical ASD population. Secondly, due to current limitations of the MVPA model used, we were unable to investigate whether autistic trait scores and OXTR genotype interactions could predict neural patterns in response to emotional faces. Thirdly, for the functional connectivity analysis we only investigated effects of four OXTR SNPs and other OXTR SNPs might have revealed additional influences on functional connectivity. Lastly, although we hypothesized the potential relevance of our findings to the “dopamine theory” or reduced reward processing in ASD we did not specifically quantify changes in dopamine or dopamine receptors in reward processing regions.

In conclusion, we used a validated implicit emotional face recognition task and dimensional approach in a large cohort of neurotypical individuals to demonstrate extensive evidence for patterns of neural responses to angry (threatening) faces in individuals which could predict autistic trait scores across “extended” face emotion processing networks involved in emotion, motivation and reward and salience but not in ‘core’ early visual processing ones. Functional connectivity changes in the identified reward (OFC - MCC) and emotion (midbrain - SMG) processing networks during angry face processing were dependent upon OXTR genotype. One of the overall implications of these findings may be that individuals with ASD process threatening face expressions, such as anger, differently and potentially in a way that could lead to inappropriate responses. Given that even neurotypical individuals show altered patterns of neural responses to threatening emotional faces in cognitive and emotion processing regions which predict their level of autistic traits this may provide a potential neural diagnostic biomarker for ASD. However, this needs to be established in future studies investigating patterns of neural processing of emotional stimuli in individuals diagnosed with ASD and it may also be important to consider the modulating influence of OXTR genotype.

### Supplementary information

Supplementary Material

## Data Availability

Behavioral data and statistical parametric maps at the group level from all fMRI analyses are available via the Open Science Framework (https://osf.io/dqyfn/). Other data can be obtained from the corresponding authors within the context of a formal data sharing agreement. Analyses were conducted using standard processing scripts in SPM12 (www.fil.ion.ucl.ac.uk/spm), the Spider toolbox (http://people.kyb.tuebingen.mpg.de/spider) and SPSS 23 (IBM Corp. Released 2016. IBM SPSS Statistics for Windows, Version 23.0. Armonk, NY: IBM Corp).
