# Supplementary material for: Patterns of neural activity in response to threatening faces are predictive of autistic traits: modulatory effects of oxytocin receptor genotype
Source: Transl Psychiatry. 2024 Mar 29;14:168. doi: 10.1038/s41398-024-02889-w (PMC10980722; doi:10.1038/s41398-024-02889-w)
Supplement: Supplementary file 1 — Supplementary Material [file 41398_2024_2889_MOESM1_ESM.docx]

***Supplementary Information***

**Patterns of neural activity in response to threatening faces are predictive of autistic traits: modulatory effects of oxytocin receptor genotype**

Xiaoxiao Zheng^1, 2#^, Feng Zhou^3#^, Meina Fu^1^, Lei Xu^4^, Jiayuan Wang^1^, Jialin Li^1^,

Keshuang Li^1^, Cornelia Sindermann^5^, Christian Montag^6^, Benjamin Becker^7^, Yang Zhan^2^*, Keith M. Kendrick^1^*

**Supplementary Methods**

**fMRI implicit emotion recognition paradigm**

Facial stimuli displaying angry, fear, happy, neutral or sad expressions (n = 10 per category, 50% male, each from different identities) were used in the event-related designed implicit emotional face recognition task. Each of the 50 facial stimuli were presented twice in two runs with a different pseudorandom sequence for all the participants. All faces were standardized into gray-scale pictures and covered with an oval mask to remove hair and other individual features using Photoshop CS6.0 (see Figure 1). All stimuli were initially rated with respect to discrimination of sex (male or female), emotion category discrimination (angry, fear, happy, neutral or sad), emotion intensity (ranging from1-9 with 1 indicating “not at all” to 9 “extremely intense”), and arousal (1-9 with 1 indicating “not aroused at all” to 9 “extremely aroused”) by an independent group of participants (n = 20, 10 males, age range = 20-22 years, mean age $\pm$ SD = 21.20 $\pm$ 0.70 years). All selected faces had high discrimination accuracy for both sex (> 90.5%) and emotion (> 88% for each face emotion).

For each trial, stimuli were presented for 2.5s following by a jittered inter-trial interval (2-5.6s, gap 0.6s). Participants were instructed to view the facial stimuli attentively and were required to indicate by a button press the sex of the face during the presentation (the face remained on screen for the full 2.5s) to ensure attentive processing (discrimination accuracy for sex = 93.46% $\pm$ 6.8%). The structure of example trials were visualized in Figure 1a. The implicit emotion recognition task paradigm was presented via E-prime 2.0 (http://www.pstnet.com/eprime.cfm, Psychology Software Tools, USA).

**Post-fMRI tests**

About forty minutes after fMRI scanning subjects completed a surprise face recognition memory test (Figure S1a) to establish if they remembered the facial stimuli presented during fMRI. During this test 100 faces (all 50 faces used in the fMRI face processing intermixed with another new 50 faces) depicting angry, happy, sad, fear, and neutral were displayed again and subjects responded if they had seen each face during the initial presentation or not. Response time (RT) and recognition accuracy were recorded. An additional explicit emotional face recognition task (Figure S1b) was subsequently implemented, and subjects were required to identify the face emotion as well as rating its emotion intensity (1-9 with 1 indicating “not at all” to 9 “extremely intense”) and their arousal effect (1-9 with 1 indicating “not aroused at all” to 9 “extremely aroused”) for the 50 emotional faces presented during fMRI.

**Genotyping and distribution of genotypes**

Genotyping was carried out as previously described (Montag et al., 2017). Briefly, DNA was extracted from participants’ buccal cells. Automated purification of genomic DNA was conducted using a MagNA Pure 96 machine and commercial extraction kits (Roche Diagnostics, Mannheim, Germany). Four common OXTR SNPs were extracted based on previous studies suggesting associations with social behavior (rs2254298, rs2268491, rs2268498, rs53576) (Feldman, Monakhov, Pratt, & Ebstein, 2016; Jurek & Neumann, 2018; Luo et al., 2015; Tops, Habel, & Radke, 2019; Uzefovsky et al., 2019; Wu et al., 2005; Yang et al., 2017; Zimmermann et al., 2018). Genotyping of the OXTR SNPs was performed by real-time polymerase chain reaction (PCR) and subsequent melting curve detection using a Cobas Z 480 Light Cycler (Roche Diagnostics, Mannheim, Germany). With the melting curve analyses, the alleles in each SNP were distinguished by different fluorescent labels of allele-specific oligonucleotide probe pairs. Simple probe assay designs from TIBMolBiol (Berlin, Germany) were used.

Subjects for whom genotype could not be reliably identified were excluded (2 subjects for rs2254298/ rs2268491, 6 subjects for rs2268498, 7 subjects for rs53576). Distribution of the 4 OXTR genotypes were in the Hardy-Weinberg equilibrium (HWE) and alleles of the SNPs were divided into two groups as in previous studies (Kou et al., 2020; Uzefovsky et al., 2019) to increase statistical power and avoid statistical inference errors due to the small sample size of certain genotypes (see Table S1) .

**MRI data acquisition and preprocessing**

MRI data were acquired on a 3T GE MR750 system (General Electric, Milwaukee, WI) located in the neuroimaging center of the University of Electronic Science and Technology of China. High-resolution whole-brain T1-weighted structural MRI data were acquired to improve normalization of the functional images using the following parameters: repetition time (TR), 6 ms; echo time (TE), minimum; flip angle, 9°; field of view (FOV), 256 ×256 mm; image matrix, 256 × 256; slice thickness, 1 mm without gap; 156 sagittal slices. Task-based fMRI data were acquired using an echo planar imaging (EPI) sequence with the following acquisition parameters: repetition time (TR), 2000 ms; echo time (TE), 30 ms; flip angle, 90°; field of view (FOV), 240 × 240 mm; image matrix, 64 × 64; slice thickness, 3.4 mm; slice gap, 0.6 mm; 39 axial slices (interleaved ascending order). OptoActive MRI headphones (http://www.optoacoustics.com/) were used to reduce acoustic noise exposure for the participants during MRI data acquisition. The task lasted for around 12 minutes (Two sessions were implemented, each session consisted of 173 volumes).

In line with our previous studies (Zheng et al., 2019; Zhou et al., 2020), functional MRI data were preprocessed using SPM12 (Statistical Parametric Mapping; <http://www.fil.ion.ucl.ac.uk/spm>) with the following steps: (1) discard the first 10 volumes of each functional time series to allow for MRI signal equilibrium and active noise cancelling by the headphones, (2) head motions corrected, (3) segment of T1 images, (4) co-registration, (5) spatial normalization (Montreal Neurological Institute (MNI) space (interpolated to 2 x 2 x 2 mm voxel size)), (6) smoothing (FWHM = 8 mm). Exclusion criteria for excessive head movement were >2.5 mm translation or >2.5° rotation.

**Supplementary Results**

**Results of correlational analyses between ASQ scores and behavioral indices using Pearson tests (uncorrected)**

***Implicit emotion recognition task.*** No significant associations between ASQ scores and emotion-specific accuracy or RT were found (Accuracy: *rs* < -0.075, *ps* > 0.260; RT: *rs* < - 0.09, *ps* > 0.175.

***Surprise face recognition memory test.*** No significant associations between ASQ scores and face memory accuracy or RT were found (Accuracy: *rs* < 0.116 *ps* > 0.082 RT: *rs* < 0.096 *ps* > 0.149.

***Explicit emotion recognition task.*** No significant associations between ASQ scores and emotion discrimination accuracy or RT were found (Accuracy: *rs* < -0.083, *ps* > 0.210 RT: *rs* < 0.113, *ps* > 0.090. Moreover, no significant associations were found between ASQ and arousal or intensity ratings across all emotion categories (Arousal: *rs* < -0.122, *ps* > 0.066; Intensity: *rs* < -0.114, *ps* > 0.085).

**Supplementary Tables**

**Table S1.** Distribution of the investigated OXTR SNPs in the current sample.

| **SNP** | **N** | **Genotypes** | **Hardy–Weinberg** | **Alleles grouped** |
| --- | --- | --- | --- | --- |
| rs2254298 | 227 | GG = 113;GA = 95;AA = 19 | χ2 = 0.024, *p* = 0.877 | A+ = 114, A- = 113 |
| rs2268491 | 227 | CC = 116;CT = 92;TT = 19 | χ2 = 2.334, *p* = 0.127 | T+ = 111, T - = 116 |
| rs2268498 | 223 | CC = 29;CT = 92;TT = 102 | χ2 = 1.283, *p* = 0.257 | C+ = 121, C- = 102 |
| rs53576 | 222 | AA = 116;AG = 87;GG = 19 | χ2 = 0.217, *p* = 0.641 | G+ =106, G- =116 |

**Note:** OXTR = oxytocin receptor; SNP = single nucleotide polymorphism.

**Supplementary Figures**

**
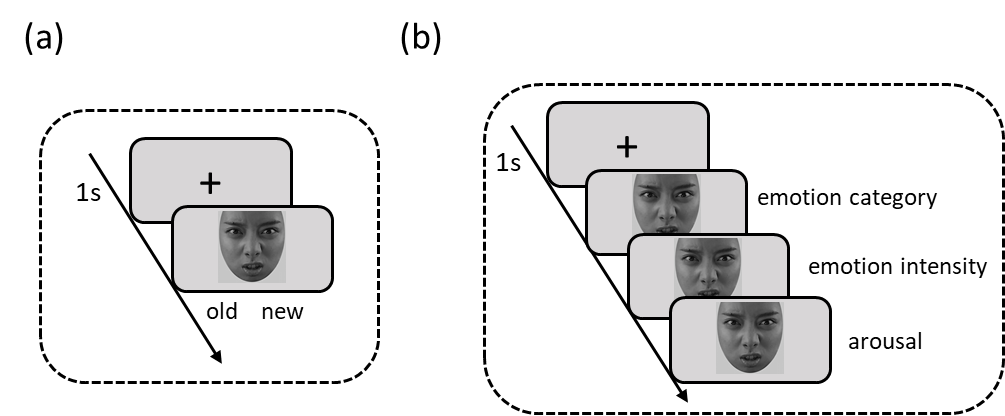
**

**Figure S1. Flowchart of the post-fMRI tests. (a)** Surprise face recognition memory test; 100 faces (all 50 faces used in the fMRI face processing intermixed with another new 50 faces) depicting angry, happy, sad, fear, and neutral were displayed again and participants responded if they had seen each face during the initial presentation or not. **(b)** Explicit emotion recognition task. 50 faces presented during fMRI scanning were displayed again and participants need to identify the face emotion as well as rating its emotion intensity (1-9 with 1 indicating “not at all” to 9 “extremely intense”) and their arousal effect (1-9 with 1 indicating “not aroused at all” to 9 “extremely aroused”).


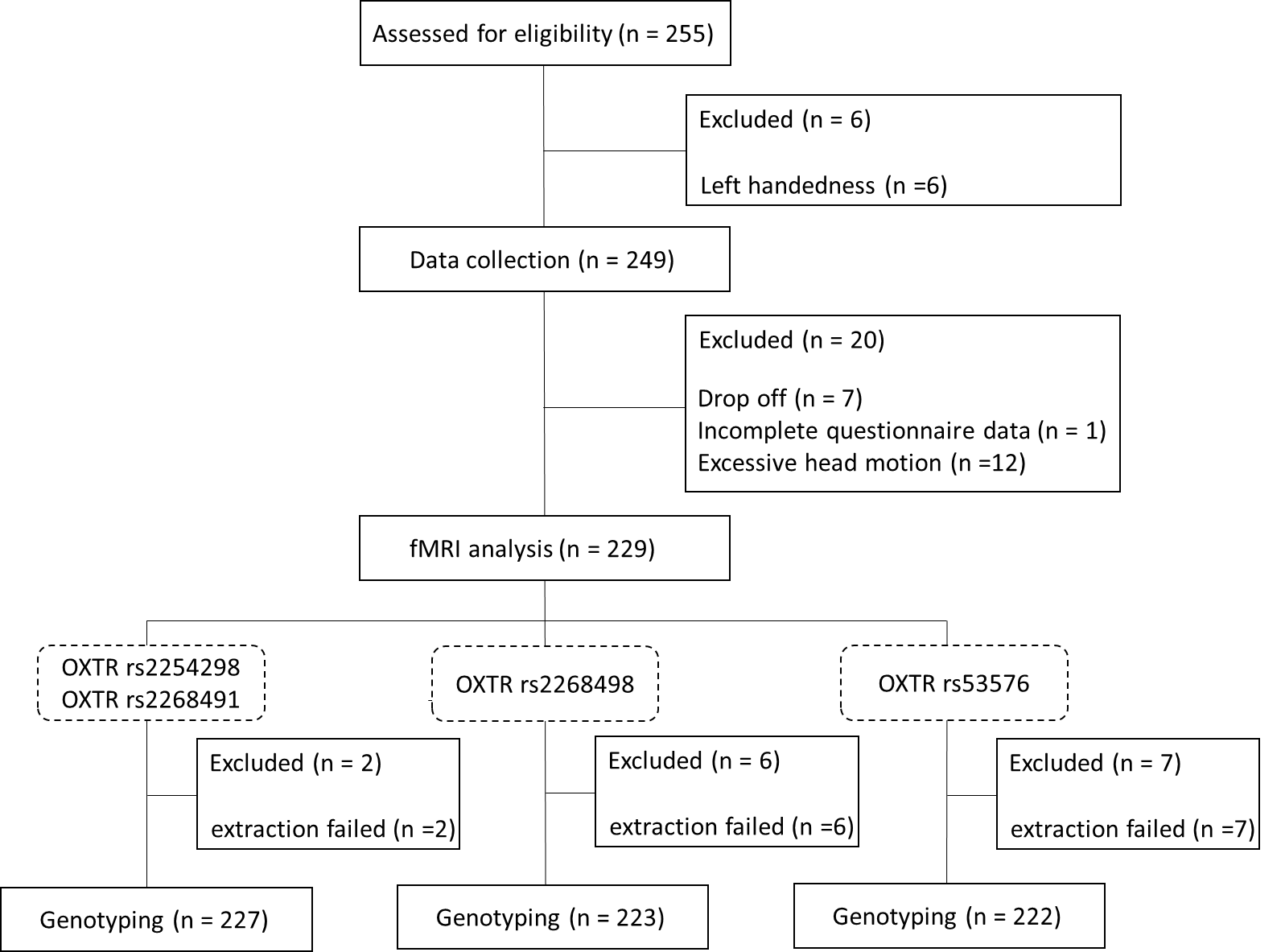


**Figure S2.** Flow diagram displaying exclusion of participant and rationale for exclusion.

**
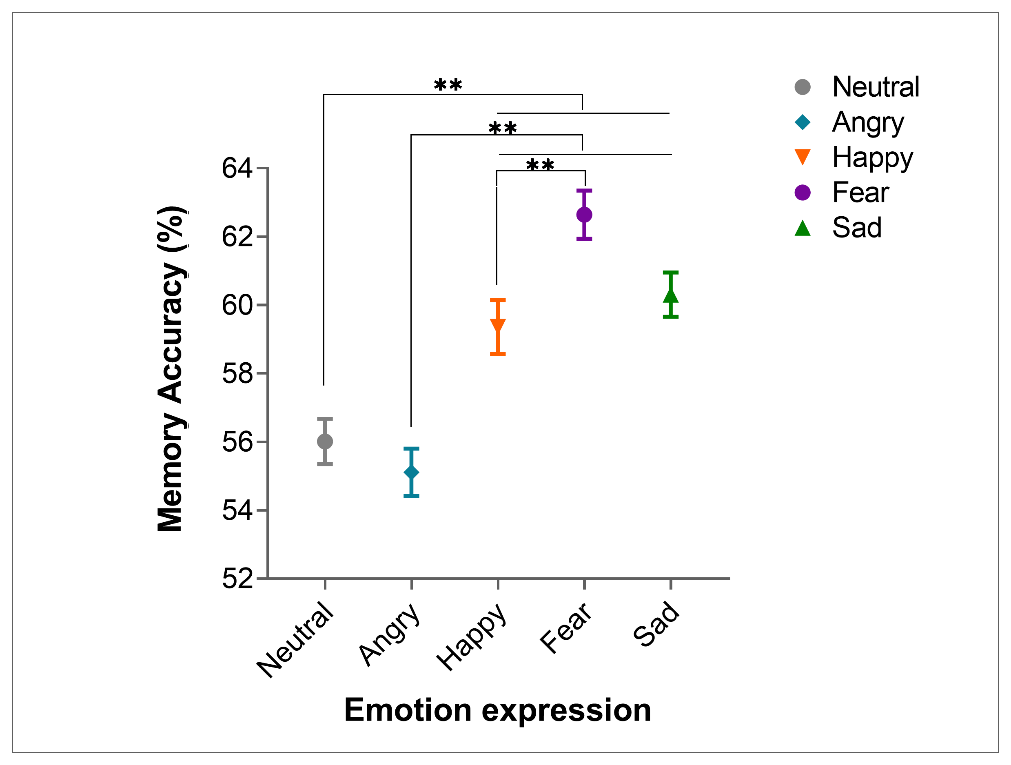
**

**Figure S3. Results of the post-fMRI surprise face recognition memory test.** The recognition memory accuracy for each emotion expression performed by participants. *^**^p* < 0.01.

**
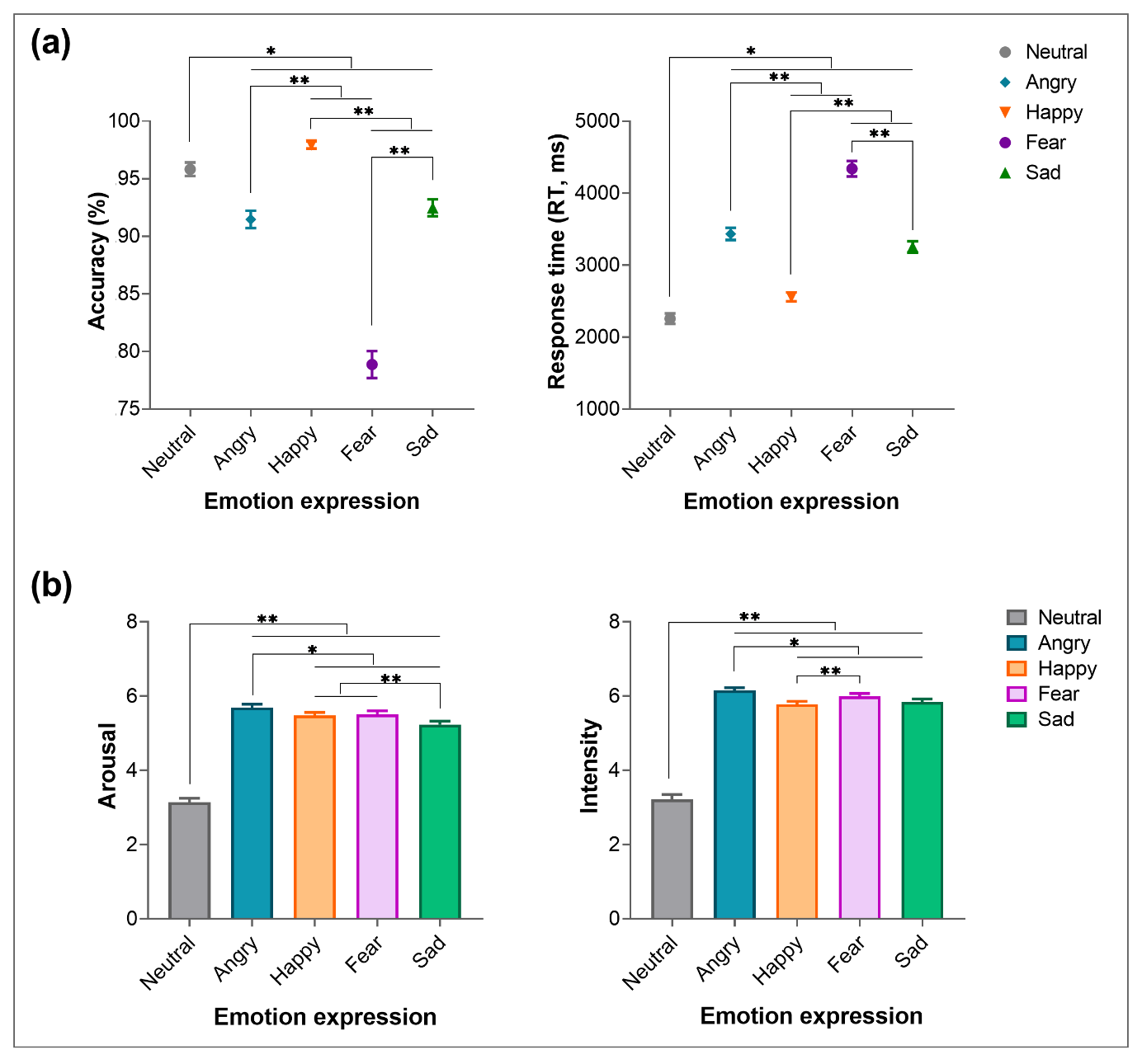
Figure S4. Results of the post-fMRI explicit emotion recognition task.** **(a)** Emotion discrimination accuracy (left panel) and response time (RT) (right panel) performed by subjects. **(b)** Ratings of arousal (left panel) and emotion intensity (right panel) given by participants. *^*^p* < 0.05, *^**^p* < 0.01.

**References**

Feldman, R., Monakhov, M., Pratt, M., & Ebstein, R. P. (2016). Oxytocin Pathway Genes: Evolutionary Ancient System Impacting on Human Affiliation, Sociality, and Psychopathology. *Biological Psychiatry*, *79*(3), 174–184. doi: 10.1016/j.biopsych.2015.08.008

Jurek, B., & Neumann, I. D. (2018). The Oxytocin Receptor: From Intracellular Signaling to Behavior. *Physiological Reviews*, *98*(3), 1805–1908. doi: 10.1152/physrev.00031.2017

Kou, J., Zhang, Y., Zhou, F., Sindermann, C., Montag, C., Becker, B., & Kendrick, K. M. (2020). A randomized trial shows dose-frequency and genotype may determine the therapeutic efficacy of intranasal oxytocin. *Psychological Medicine*, 1–10. doi: 10.1017/S0033291720003803

Luo, S., Ma, Y., Liu, Y., Li, B., Wang, C., Shi, Z., … Han, S. (2015). Interaction between oxytocin receptor polymorphism and interdependent culture values on human empathy. *Social Cognitive and Affective Neuroscience*, *10*(9), 1273–1281. doi: 10.1093/scan/nsv019

Montag, C., Sindermann, C., Melchers, M., Jung, S., Luo, R., Becker, B., … Kendrick, K. M. (2017). A functional polymorphism of the OXTR gene is associated with autistic traits in Caucasian and Asian populations. *American Journal of Medical Genetics Part B: Neuropsychiatric Genetics*, *174*(8), 808–816. doi: 10.1002/ajmg.b.32596

Tops, S., Habel, U., & Radke, S. (2019). Genetic and epigenetic regulatory mechanisms of the oxytocin receptor gene (OXTR) and the (clinical) implications for social behavior. *Hormones and Behavior*, *108*, 84–93. doi: 10.1016/j.yhbeh.2018.03.002

Uzefovsky, F., Bethlehem, R. A. I., Shamay-Tsoory, S., Ruigrok, A., Holt, R., Spencer, M., … Baron-Cohen, S. (2019). The oxytocin receptor gene predicts brain activity during an emotion recognition task in autism. *Molecular Autism*, *10*(1), 12. doi: 10.1186/s13229-019-0258-4

Wu, S., Jia, M., Ruan, Y., Liu, J., Guo, Y., Shuang, M., … Zhang, D. (2005). Positive Association of the Oxytocin Receptor Gene (OXTR) with Autism in the Chinese Han Population. *Biological Psychiatry*, *58*(1), 74–77. doi: 10.1016/j.biopsych.2005.03.013

Yang, S., Dong, X., Guo, X., Han, Y., Song, H., Gao, L., … Zhang, X. (2017). Serum Oxytocin Levels and an Oxytocin Receptor Gene Polymorphism (rs2254298) Indicate Social Deficits in Children and Adolescents with Autism Spectrum Disorders. *Frontiers in Neuroscience*, *11*, 221. doi: 10.3389/fnins.2017.00221

Zheng, X., Luo, L., Li, J., Xu, L., Zhou, F., Gao, Z., … Kendrick, K. M. (2019). A dimensional approach to jealousy reveals enhanced fronto-striatal, insula and limbic responses to angry faces. *Brain Structure and Function*, *224*(9), 3201–3212. doi: 10.1007/s00429-019-01958-x

Zhou, F., Li, J., Zhao, W., Xu, L., Zheng, X., Fu, M., … Becker, B. (2020). Empathic pain evoked by sensory and emotional-communicative cues share common and process-specific neural representations. *ELife*, *9*, e56929. doi: 10.7554/eLife.56929

Zimmermann, J., Deris, N., Montag, C., Reuter, M., Felten, A., Becker, B., … Markett, S. (2018). A common polymorphism on the oxytocin receptor gene (rs2268498) and resting-state functional connectivity of amygdala subregions—A genetic imaging study. *NeuroImage*, *179*, 1–10. doi: 10.1016/j.neuroimage.2018.06.014
